# Reidel's Thyroiditis, a Diagnostic and Management Challenge: A Case Report and Review of the Literature

**DOI:** 10.1155/2021/5185259

**Published:** 2021-10-12

**Authors:** Yassine Er-Rahali, Mohammed Massine El Hammoumi, Jad Issouani, Colna Antonio Nfad, Souad El Moussaoui, El Hassane Kabiri, Ahmed Anass Guerboub

**Affiliations:** ^1^Endocrinology Department, Mohammed V Military Academic Hospital, Faculty of Medicine and Pharmacy, Mohammed V-Souissi University, Rabat, Morocco; ^2^Thoracic Surgery Department, Mohammed V Military Academic Hospital, Faculty of Medicine and Pharmacy, Mohammed V-Souissi University, Rabat, Morocco

## Abstract

Riedel's thyroiditis is a very rare inflammatory condition. It affects not only the thyroid gland but also the adjacent vital structures. It may also be associated with different forms of systemic fibrotic disorders. The exact etiology is unknown, but currently, the most favorable opinion is that it is a localized form of the systemic fibrotic process. We report the case of a 38-year-old woman, presented with a 10-month history of progressive hypothyroidism, dysphonia, and dysphagia. A Doppler ultrasound study revealed massive thyroid enlargement with multiple Eu TIRADS 3 and 4 nodules. Fine needle aspiration was noncontributive on two occasions. A hard subtotal thyroidectomy was performed. Pathological study confirmed Riedel's thyroiditis with the presence of IgG4 antibodies in immunohistochemistry. The patient was successfully treated with levothyroxine replacement and corticosteroid therapy with rapid resolution of obstructive symptoms. The case descriptions highlight the diagnostic challenge of this disease, describe the response to surgical management and corticosteroid therapy, and give a short review of the subject.

## 1. Introduction

Riedel's thyroiditis (RT) is a rare fibrosclerotic condition affecting the thyroid gland. It is characterized by thyroid parenchyma replacement with fibrous tissue. It was first described by Bernhard Riedel in 1896 [1]. Until now, there have been almost 200 cases reported in the literature. This sclerotic process may not only be limited to only the thyroid but also invades the surrounding vital structures such as vessels, nerves, trachea, esophagus, and parathyroid that leads to compressive symptoms and endocrine abnormalities [2]. It is a rare condition, with a prevalence of 1 per 100,000 inhabitants, which affects more frequently women between 30 and 50 years of age. RT accounts for less than 0.06% of patients undergoing thyroidectomies [3]. The precise etiology of this disorder is still unclear. An association with systemic fibrotic processes, autoimmune diseases, and more recently with diseases from the spectrum of excessive immunoglobulin G type 4 (IgG4) has been described [4]. We report the case of a 38-year-old woman presented with clinical features initially suggestive of malignant goiter, which turned out to be Riedel's thyroiditis.

## 2. Case Report

**Figure 1:**
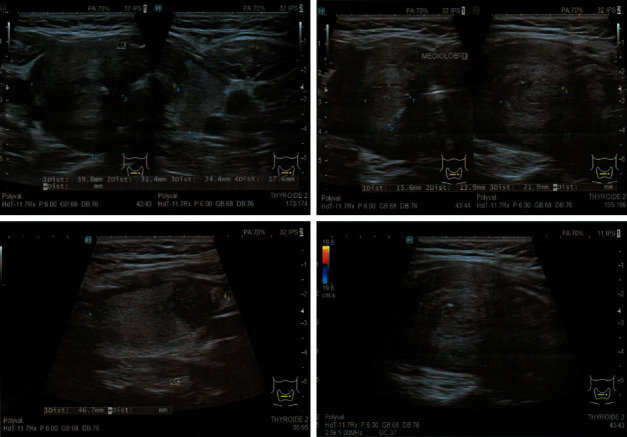
Patient's cervical ultrasound showing a hypovascularized nodular goiter.

**Figure 2:**
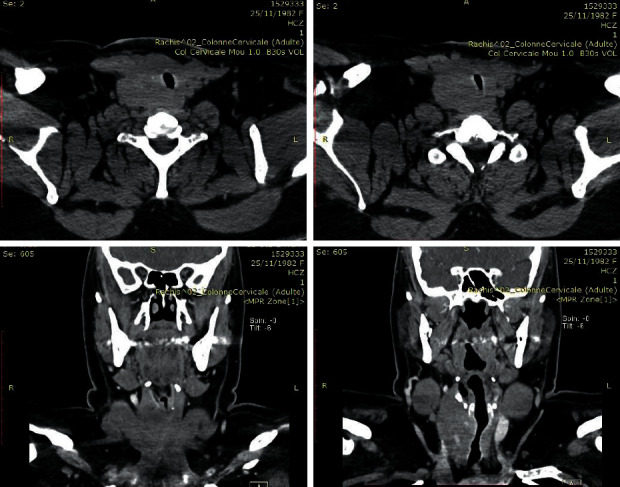
CT image in transverse and coronal sections showing a large goiter with tracheal deviation and stenosis.

We report the case of a 38-year-old woman who came to the endocrinology department with a 10-month history of progressive hypothyroidism and an enlargement at the base of her neck. She had noted dysphonia, dyspnea, and progressive difficulty in swallowing solid food. Thyroid-stimulating hormone was normal under on L-thyroxin replacement therapy. Antithyroglobulin and antiperoxidase antibodies were negative. Parathormone and calcemia levels were normal before surgery (Table 1). A Doppler ultrasound study of the woman's neck revealed a massive thyroid enlargement with multiple Eu TIRADS 3 and 4 nodules (Figure 1). CT scan showed a large nodular goiter with tracheal deviation and stenosis (Figure 2). Fine needle aspiration was noncontributive on two separate occasions (Bethesda I).

The patient was referred to surgery for thyroidectomy. Intraoperative findings revealed a goiter with a hard stony consistency, adhesive to the visceral planes suggestive of huge malignancy. Peroperative pathological examination of a specimen suspected malignant thyroid disease. The surgical team performed despite a subtotal thyroidectomy with placement of a temporary tracheostomy. The definitive pathological study confirmed surprising diagnosis of Riedel's thyroiditis with the presence of IgG4 antibodies in immunohistochemistry.

Evolution was marked by progressive dyspnea. The patient was put on short courses of corticosteroids with a transitory symptom improvement. The patient was admitted to our department 5 months after surgery because of COVID-19 pandemic. On physical examination, the patient presented signs of hypocalcemia with dyspnea. Laboratory tests confirmed hypoparathyroidism treated by substitutive therapy (alfacalcidol + calcium). A cervico-thoraco-abdominal CT scan revealed a right thyroid infiltrating process and plunging into the upper mediastinum. This process encases the right primary carotid artery up to its bifurcation. A partial thrombosis of the right internal jugular vein was noted. No fibrous lesion elsewhere. The orbital CT scan was normal. The serum level for immunoglobulin G4 (IgG4) subclass had been normal.

The patient was successfully treated with oral levothyroxine replacement (100 *μ*g per day) high-dose corticosteroid therapy (60 mg/day) for 1 month. Internal jugular vein thrombosis was treated successfully with the 3-month use of enoxaparin.

On follow-up, dyspnea had resolved. A gradual reduction of corticosteroid doses was performed. Currently, the patient is on 7.5 mg/d of prednisolone. The cervical ultrasound shows a stable right thyroid residue.

## 3. Discussion

Riedel's thyroiditis is extremely rare. The incidence is now estimated to be 1 in 100,000, with higher incidence in women. It is most frequent in middle age adults (30–50 years) [2, 3]. There are multiple hypotheses regarding its pathogenesis, but its exact etiology remains uncertain. Autoimmune etiology is the most likely hypothesis. Some authors consider Riedel's thyroiditis as a part of multifocal fibrosclerosis, relating to the group of IgG4 sclerosing diseases. [5].

Patients with RT usually present compressive symptoms related to fibrosis, such as hoarseness, aphonia, dysphagia, and/or dyspnea. Physical exam often finds a firm, fixed thyroid, which suspects a malignant tumor. If neck surgery is pursued, like in our case, surgeons will find a large fibrotic mass involving the thyroid and perithyroid tissue without an obvious tissue plane [6]. As the fibroinflammatory process progresses, it can affect the thyroid and parathyroid glands, leading to hypothyroidism and/or hypoparathyroidism. Evidence collected over time shows that RT-associated hypothyroidism occurs in 25–80% of patients [7].

The thyroid ultrasound typically finds a diffuse hypoechogenous thyroid that seems to be hypovascular on color Doppler. A thyroid mass or node may also be found, as in our patient's case. Additional imaging techniques have also been performed: ultrasound elastography revealed increased gland stiffness. 18-Fluoro-deoxyglucose PET/CT showed increased uptake by the lesion, inconstantly decreased by corticoids, and 99mTc thyroid scintigraphy showed poor radioactive uptake. MRI results vary widely among authors. Although numerous imaging techniques have been used, clinicians remain unable to differentiate Riedel's thyroiditis from other thyroid pathologies, including neoplasms [8]. Cytology is not always diagnostic in RT and may fail to prove the invasive nature of fibrosis [9]. The diagnosis of RT is most commonly suggested after pathologic evaluation of resected thyroid tissue. Findings like storiform fibrosis and occlusive phlebitis argue for RT, and, in the case of IgG4 involvement, elevation of IgG4 positive plasma cells with an IgG-4 ratio >40% [10].

Because of its low incidence, no guidelines or large clinical studies exist for the optimal management of Riedel's thyroiditis. Most authors agree that therapy must focus on treating hypothyroidism and managing fibrosclerotic manifestations [11]. Systemic steroids are the first-line therapy, decreasing the size and hardness of the goiter in only a few patients. Prolonged treatment is often required due to the risk of losing the improvement achieved once the doses are reduced. Tamoxifen is another agent used successfully, at doses of 10–20 mg twice a day. Significant reduction in goiter size has been reported in patients who have not responded to steroids previously [12].

Other potential therapies exist, but may be less well studied. There are a few reports of the use of low-dose radiation for the treatment of RT, but no systematic evaluation of this therapy exists to date. Mycophenolic acid, the active metabolite of mycophenolate, induces inhibition of T- and B-cell production, thus reducing the spread of antibody levels [13]. Rituximab has been reported to have favorable effects in IgG4-related systemic disorders and in some cases of RT [14]. As far as surgical management is considered, the indications are essentially two: sample for histopathological diagnostic confirmation and relieving compression or obstruction in the trachea or esophagus. The complete resection of the gland is not recommended due to the high risk of damaging nearby structures such as neck vessels, the parathyroid, and recurrent laryngeal nerves because of the lack of resection margins [11].

## 4. Conclusion

Reidel's thyroiditis is a very rare form of fibrous destruction of thyroid parenchyma, which leads to thyroid dysfunction and progressive airway and digestive tract obstruction. Glucocorticoids are still the mainstay of therapy. The need of consensual guidelines to better manage this rare disease becomes mandatory to prevent associated complications.

## Figures and Tables

**Table 1:** Patient's laboratory results.

Laboratory tests	Patient values		Normal range
TSH	0.89 *μ*U/ml		0.40–4.00
Free T4	1.35 ng/dl		0.70–1.48
TPO ab	10.2 U/l		<34
Tg ab	1.57 U/ml		0.20–4.11
Calcium	Before surgery	91 mg/l	80–105
	Follow-up	77 mg/l	
PTH	Before surgery	66 pg/ml	15–68
	Follow-up	21 pg/ml	
Serum-IgG4	0.071 g/L		0.039–0.864

TSH: thyroid stimulating hormone; TPO ab: thyroid peroxidase antibodies; Tg ab: thyroglobulin antibodies; PTH: parathyroid hormone; IgG4: subclass 4 of immunoglobin G.
